# The Week's Work

**Published:** 1911-02-11

**Authors:** 


### The Week's Work.

### LEEDS GENERAL INFIRMARY.

We have received a copy of a booklet of the work in progress for this Infirmary from 1767 to 1911, issued in support of the appeal for £150,000, to which we referred in our issue of the 4th instant. It is properly pointed out that the majority of the existing wards require modernising and reconstruction as to their sanitary arrangements; that four new operation theatres are needed, and we should say absolutely essential to the welfare of the patients and the staff who are responsible for their treatment and care; that two additional wards must be provided, the population of Leeds having increased from 207,000 when the present hospital was approved to nearly half a million persons. The number of beds in the hospital has only been increased by 125 since 1864, and many more are absolutely essential for the protection of the poorer inhabitants. As we pointed out in our issue of January 29, 1910, the pathological department, including the mortuary and its adjuncts, urgently calls for reconstruction and addition, and much additional accommodation for nurses has become a paramount necessity in order to bring the nursing staff up to modern requirements. These and other minor matters will entail a large outlay, and £150,000 is not too much to ask for in the circumstances. Mr. Charles Lupton, the treasurer, is an experienced and able economist in hospital management, who has ever shown the way to save expenditure whilst securing the maximum of efficiency. In such circumstances we are glad to notice that, although the movement to raise the necessary funds was only started by the Lord Mayor on the 26th ultimo, it has already produced about half the required sum from a relatively few contributors, so that the success of the appeal seems assured. How could it be otherwise in a city like Leeds, which prides itself upon its intelligence and critical acumen? We should say that it cannot be questioned that never has a hospital appeal for £150,000 been made with greater justification nor by a Hospital Committee which better deserves the confidence of those who are asked to provide the necessary funds. We hope, therefore, this appeal may prove a record in the sense that the whole £150,000 may be subscribed within two months, *i.e.* on or before March 31 next.

### THE SUNDERLAND INFIRMARY'S PETITION.

The Sunderland Infirmary has decided to petition His Majesty to allow it to assume the title Royal. Our readers will remember how the recent case of the Royal Portsmouth Hospital, which was recorded in The Hospital of January 21, page 516, illustrated the truth of our previous contention that permission to use the prefix Royal "has always implied and expressed the good work done by the institution in question." That being so, the record of work attached, under seven headings, to the petition of the Sunderland Infirmary becomes of much importance. What, the advisers of His Majesty will ask themselves, does this great hospital claim to have done? In the first place it claims to have been a pioneer in the starting of workmen's contributions, and in granting free admission to patients by the abolition of the privileges of the governors. Again, it is one of the oldest hospitals in the county of Durham, having been founded in 1794, and for eighteen years has had a convalescent home for the after-care of patients. Apart from this the Sunderland Infirmary has a children's hospital built for 50 patients, which will be opened when the further £2,000 a year required for its maintenance is forthcoming. The efforts made to raise this sum were applauded in The Hospital of October 22 (page 119), and we cannot but think that the additional argument most likely to influence His Majesty to answer the petition favourably would be the speedy raising of the money required. Mr. T. R. Blumer must also have felt at the annual meeting that the petition would read more convincingly if it had been accompanied by the often postponed resuscitation of the present old administration block. As chairman of the Finance Committee, Mr. Blumer has now a fine opportunity of bringing forward the need for a new building to the notice of the miners and the hospital's supporters generally. Sunderland Infirmary assuredly would gain the title of Royal as soon as this is done. As it is, the fine work of the institution is hampered and may not yet receive the Royal favour it asks for on this account.

### WORKERS' HOSPITAL FUND AT CAMBRIDGE.

All the hopeful prophecies concerning this fund are being fulfilled. It was founded last summer, and The Hospital has been recording its quarterly career since then. The first quarter realised a sum total of £166, as may be seen in our issue of October 22; and the second quarter, under Colonel T. W. Harding's presidency, has raised this sum to £317. That is a very fine achievement, and justifies Colonel Harding's hope that the Workers' Fund at Cambridge my enable the extension of Addenbrooke's to be undertaken by becoming the chief power in the collection of the £15,000 necessary for this purpose. The Workers' Fund, indeed, with its 82 centres of collection, has produced already a far-reaching effect, for there is no doubt that the response it was receiving encouraged the Mayor of Cambridge to make his appeal for the extension fund which will make the rebuilt wing of Addenbrooke's the University town's memorial to King Edward. This should encourage the fund still further, and we are happy to have recorded its progress from the foundation to its present stage of promising growth.

### THIS WEEK'S DRUG MARKET.

Business in the drug market is good, and the general tendency of prices is in an upward direction. A number of articles have advanced in value. Carbolic acid is 50 per cent. dearer; such a substantial advance in the price of this article is altogether unusual, but the cause is the increased demand due to the plague in Manchuria. Opium has increased in value, and both morphine and codeine are dearer in consequence, the price of the latter alkaloid having been advanced 10d. per ounce. Quicksilver has been advanced 10s. per bottle, and makers of calomel, corrosive sublimate, and other salts of mercury have added one penny per lb. to the quotations for these salts. A good business has been done in camphor at advancing rates, both Japanese and English refined being dearer. Oil of peppermint is dearer, and still inclined to advance. Menthol is also steadily advancing in price. Buchu leaves are again tending dearer. The higher value of ergot of rye is well maintained. Potassium bromide and other salts of bromine are not unlikely to be advanced in price. Hydrastis canadensis is dearer. A further rise in the value of cocaine is not unexpected. Oil of almonds and oil of cloves may probably be dearer. Definite reports from Norway as to the cod-fishings are still awaited, but in the meantime the price of cod liver oil is well maintained with an inclination to advance. It is a long time since there have been so many drugs attracting the attention of buyers.

